# Frequency of Adverse Events after Vaccination with Different Vaccinia Strains

**DOI:** 10.1371/journal.pmed.0030272

**Published:** 2006-08-22

**Authors:** Mirjam Kretzschmar, Jacco Wallinga, Peter Teunis, Shuqin Xing, Rafael Mikolajczyk

**Affiliations:** 1 Department of Infectious Diseases Epidemiology, Rijksinstituut voor Volksgezondheid en Milieu/National Institute for Public Health and the Environment, Bilthoven, Netherlands; 2 School of Public Health, University of Bielefeld, Bielefeld, Germany; 3 Computerization and Methodological Consultancy Unit, Rijksinstituut voor Volksgezondheid en Milieu/National Institute for Public Health and the Environment, Bilthoven, Netherlands; Vaccine and Gene Therapy Institute, United States of America

## Abstract

**Background:**

Large quantities of smallpox vaccine have been stockpiled to protect entire nations against a possible reintroduction of smallpox. Planning for an appropriate use of these stockpiled vaccines in response to a smallpox outbreak requires a rational assessment of the risks of vaccination-related adverse events, compared to the risk of contracting an infection. Although considerable effort has been made to understand the dynamics of smallpox transmission in modern societies, little attention has been paid to estimating the frequency of adverse events due to smallpox vaccination. Studies exploring the consequences of smallpox vaccination strategies have commonly used a frequency of approximately one death per million vaccinations, which is based on a study of vaccination with the New York City Board of Health (NYCBH) strain of vaccinia virus. However, a multitude of historical studies of smallpox vaccination with other vaccinia strains suggest that there are strain-related differences in the frequency of adverse events after vaccination. Because many countries have stockpiled vaccine based on the Lister strain of vaccinia virus, a quantitative evaluation of the adverse effects of such vaccines is essential for emergency response planning. We conducted a systematic review and statistical analysis of historical data concerning vaccination against smallpox with different strains of vaccinia virus.

**Methods and Findings:**

We analyzed historical vaccination data extracted from the literature. We extracted data on the frequency of postvaccinal encephalitis and death with respect to vaccinia strain and age of vaccinees. Using a hierarchical Bayesian approach for meta-analysis, we estimated the expected frequencies of postvaccinal encephalitis and death with respect to age at vaccination for smallpox vaccines based on the NYCBH and Lister vaccinia strains. We found large heterogeneity between findings from different studies and a time-period effect that showed decreasing incidences of adverse events over several decades. To estimate death rates, we then restricted our analysis to more-recent studies. We estimated that vaccination with the NYCBH strain leads to an average of 1.4 deaths per million vaccinations (95% credible interval, 0–6) and that vaccination with Lister vaccine leads to an average of 8.4 deaths per million vaccinations (95% credible interval, 0–31). We combined age-dependent estimates of the frequency of death after vaccination and revaccination with demographic data to obtain estimates of the expected number of deaths in present societies due to vaccination with the NYCBH and Lister vaccinia strains.

**Conclusions:**

Previous analyses of smallpox vaccination policies, which rely on the commonly assumed value of one death per million vaccinations, may give serious underestimates of the number of deaths resulting from vaccination. Moreover, because there are large, strain-dependent differences in the frequency of adverse events due to smallpox vaccination, it is difficult to extrapolate from predictions for the NYCBH-derived vaccines (stockpiled in countries such as the US) to predictions for the Lister-derived vaccines (stockpiled in countries such as Germany). In planning for an effective response to a possible smallpox outbreak, public-health decision makers should reconsider their strategies of when to opt for ring vaccination and when to opt for mass vaccination.

## Abstract 2

Analysis of historical data for adverse events suggests that the commonly assumed number of one death per million vaccinations is inaccurate. Large differences between different vaccinia strains used should be taken into account when mass vaccinations are considered.

## Editors' Summary

**Background.**

For thousands of years, smallpox was one of the world's most-feared diseases. This contagious disease, caused by the variola virus, historically killed about 30 percent of the people it infected. Over the centuries, it probably killed more people than all other infectious diseases combined, but it was also the first disease to be prevented by vaccination. In 1796, the English physician Edward Jenner rubbed pus from the spots of a milkmaid with cowpox into scratches on a young boy's arm; according to folklore, people who caught cowpox, a related but mild disease of cows, were protected against smallpox. Six weeks later, after a mild bout of cowpox, when the boy was challenged with pus from a smallpox patient, he did not develop smallpox. This vaccination procedure was later refined so that people were inoculated with pure preparations of live vaccinia virus, which is closely related to the smallpox and cowpox viruses, and by 1979 a global vaccination campaign had totally eradicated the disease.

**Why Was This Study Done?**

Smallpox vaccination has some adverse effects. In particular, vaccinia virus occasionally infects the brain. This so-called post-vaccination encephalitis can cause permanent brain damage and, it has been estimated, kills one vaccinee in every million. Consequently, as smallpox became rarer, the dangers of vaccination began to outweigh its benefits. Routine smallpox vaccination stopped in the US in 1972, and in 1980 the World Health Organization recommended that all countries stop vaccination. Now, however, there are fears that smallpox may be used for bioterrorism. If this did happen, exposed individuals and their contacts, possibly even whole populations, would have to be vaccinated as quickly as possible (very few people now have strong immunity to smallpox). Many countries have stockpiles of smallpox vaccines for this eventuality, but these contain different vaccinia virus strains. In this study, the researchers examined historical data to discover whether these strains differ in their potential to cause encephalitis and death. This information should help public-health officials plan their vaccination strategies in response to a bioterrorism attack with smallpox.

**What Did the Researchers Do and Find?**

The researchers collected data from published studies on smallpox vaccination and adverse events from several countries from the late 1950s onwards. They then used these data to extrapolate how often the different vaccinia strains might cause encephalitis and death if they were used today in vaccination programs. They estimate that vaccinating with the New York City Board of Health (NYCBH) strain, which is stockpiled in the US, might cause 2.9 cases of post-vaccination encephalitis and 1.4 deaths per million vaccinated individuals. In contrast, the Lister strain, which is stockpiled in many European countries, might cause 26.2 cases of post-vaccination encephalitis and 2.5 deaths per million vaccinees. For both strains, vaccination of children younger than 1 year old would cause the highest death rate, and individuals being re-vaccinated would be less likely to die than those being vaccinated for the first time. Finally, the researchers use their figures to estimate that about ten people would die if mass vaccination with the NYCBH strain were used in the Netherlands (population 16 million), whereas 55 people would die if the Lister strain were used.

**What Do These Findings Mean?**

The data used in this study are of variable quality, so the figures calculated by the researchers are only estimates. For instance, given the scatter of the original data, mass vaccination in the Netherlands with the Lister strain might cause anywhere between seven and nearly 200 deaths. However, the study clearly suggests that more serious adverse events would occur after vaccination with the Lister strain than after vaccination with the NYCBH strain. It also indicates that even in the US, where the NYCBH vaccine strain is stockpiled, previous analyses of the effects of vaccination in response to a bioterrorist attack have probably underestimated how many people might die from post-vaccination encephalitis. Public-health decision makers should incorporate these new estimates into their planning for a smallpox outbreak. These increased estimates of adverse events after vaccination might, for example, make mass vaccination with the Lister strain of vaccinia virus less acceptable. Instead, public-health officials might decide to rely on vaccination of only the people directly exposed to released smallpox virus and their close contacts (ring vaccination) to contain a smallpox outbreak.

**Additional Information.**

Please access these Web sites via the online version of this summary at http://dx.doi.org/10.1371/journal.pmed.0030272.

- World Health Organization, information on smallpox and preparedness in the event of a smallpox outbreak
- MedlinePlus encyclopedia entry on smallpox
- US National Institute of Allergy and Infectious Diseases, patient fact sheet on smallpox
- US Centers for Disease Control and Prevention, information for patients and professionals on smallpox
- Wikipedia page on smallpox (note that Wikipedia is a free online encyclopedia that anyone can edit)
- Wellcome Library MedHist, links to information on the history of smallpox vaccination

## Introduction

With concerns rising that the smallpox virus could be used in a bioterrorist attack, many countries have been planning vaccination programs in case an outbreak of smallpox occurs. Most of the vaccines stockpiled are first-generation vaccines, as were used during the World Health Organization (WHO) smallpox-eradication campaign. Considering the large number of unvaccinated people in present societies, the question arises about how many people would be expected to have serious adverse events, such as postvaccinal encephalitis (PVE [1,2]) or death, after smallpox vaccination. Because PVE had a case-fatality rate of approximately 25%–30%, with 16%–30% of survivors having permanent neurological damage [1,2], the WHO promoted the switch to the Lister strain of vaccinia virus for vaccine production in the 1950s. It has been shown that vaccinia strains differ in their pathogenicity in animals [3] and that vaccinia strains that are more pathogenic in animals may cause a higher rate of postvaccinal complications in humans after vaccination [4].

Recent experience during a smallpox vaccination campaign in the US demonstrated clearly the importance of precise knowledge of the frequency of adverse events after vaccination. In a two-pronged program, more than 40,000 civilians and more than 700,000 military personnel were vaccinated between December 2002 and January 2005 [5]. The vaccination program for the military and civilian personnel of the US Department of Defense is ongoing, and, as of April 2006, the number of people screened exceeds 1,090,000, with more than 1 million having been vaccinated since December 2002 (J. Grabenstein, personal communication). The frequency of adverse events that was anticipated on the basis of historical data was lower than expected. However, a higher-than-anticipated number of vaccination-related myopericarditis cases led to much publicity of and controversy about the program [6]. Although the occurrence of vaccination-related adverse events cannot be precluded, this experience shows the importance of using the knowledge we have from previous vaccination campaigns during planning for future campaigns [7]. Precise estimates of the frequencies of adverse events and their credible intervals (CIs) are essential for defining thresholds for stopping or switching vaccination programs.

Mathematical modeling has played a central role in the development of plans to respond to a smallpox outbreak [8–13], because it provides a tool to rigorously test the effects of different vaccination strategies and analyze the influence of various interventions in a situation in which there is no naturally occurring infection. In many of these modeling studies, estimates of the number of deaths associated with a smallpox vaccination campaign were computed on the basis of the commonly cited mortality rate of one death per million vaccinations that was derived from the surveys by Lane et al. [14] and Neff et al. [15]. Recently, US studies about the adverse effects of smallpox vaccination with the New York City Board of Health (NYCBH) vaccinia strain that were published by Lane et al. [14,16], Neff et al. [15,17], and others were reviewed by Aragón et al. [18].

However, model findings based on the adverse effects of the NYCBH strain might not hold for the Lister strain, which is stockpiled in Great Britain, Germany, the Netherlands, and other European countries. Even less is known about the frequency of adverse events after vaccination with other vaccinia strains that might still be in use in Asian countries. An analysis of the existing data on the frequency of adverse events after vaccination with different vaccinia strains can provide some evidence of the variability between strains. At present, efforts are under way to develop third-generation vaccines based on attenuated strains of vaccinia virus, such as modified vaccinia Ankara (MVA) and LC16m8 [19,20]. The latter strain, which is based on the Lister strain and is currently licensed in Japan for use in smallpox vaccine, was administered to more than 100,000 infants during 1973–1975 without serious adverse events and is viewed as a serious candidate for a third-generation vaccine. Although there are many reports scattered in the literature on the adverse effects of smallpox vaccination with vaccinia strains other than NYCBH, those studies are often difficult to access and published in languages other than English.

In our study, we collected data from reports involving a variety of countries, periods, and age groups on vaccinations with and adverse events due to different vaccinia strains. On the basis of these data, we conducted a meta-analysis using Bayesian methods and obtained estimates for the frequency of adverse events after smallpox vaccination with respect to age and vaccinia strain. Here, we report age-related and strain-related findings on the occurrence of PVE and death after primary vaccination and revaccination with vaccinia virus. We combined age-dependent estimates with demographic data about the age structure of a population targeted for vaccination to derive estimates and CIs of the rate of adverse events, including death, to be expected during a targeted smallpox vaccination program.

### Data Sources

We conducted a systematic search of the literature using Medline and the social medicine and public health database that is located at the German Institute of Medical Documentation and Information. Furthermore, we iteratively scanned references cited in each of the identified articles for any additional studies. For data specifically about Germany, we searched the “Bundesgesundheitsblatt” (a German medical journal) from 1959 to 1985. We extracted data about numbers of primary vaccination and revaccination, age groups, strains, and frequencies of adverse events. In addition to the studies reviewed by Aragón et al. [18], we were able to extract a considerable amount of information about vaccinations in Germany, Austria, Sweden, the UK, France, the former Soviet Union, and the Netherlands (see Table 1). There was huge variation in the detail of reporting between the various studies. For the analysis, we categorized the studies according to whether the following types of information were reported: numbers of primary vaccination and revaccination, age at the time of primary vaccination and revaccination, types and frequencies of adverse events, age at which adverse events occurred, vaccinia strain used, and the time of vaccination.

**Table 1:**
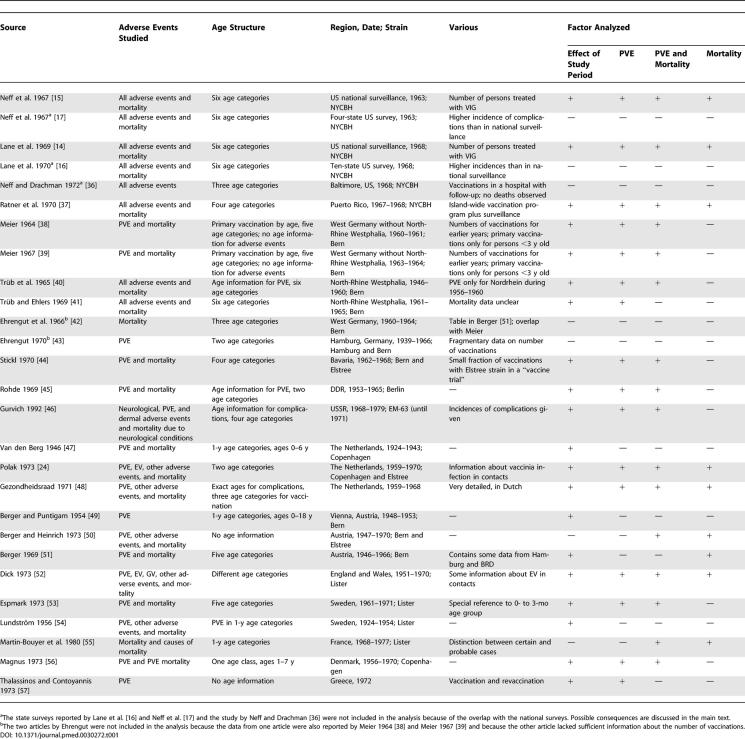
Primary Literature Used for Extracting Data about Incidence of Adverse Events after Smallpox Vaccination

Articles that did not clearly distinguish between primary vaccination and revaccination were excluded from our analysis. For the analysis of the effect of age on the frequency of adverse events, we included only studies that reported both age at vaccination and age at which adverse events occurred (although broad age categories were often used). Some studies were excluded because the reporting time and study population overlapped those of other studies. Of the US studies, we chose to use the national surveillance data reported by Lane et al. [14] and Neff et al. [15] instead of data from the state surveys. The reason for this decision was that the national data are believed to be more reliable, because they were better validated (D. Henderson, personal communication). The state reports included large numbers of trivial rashes and other minor adverse events and were not designed to document valid rates of serious events, such as PVE or death [21]. So, although the national reports may suffer from some under-reporting, the cases that they report are better validated.

The vaccinia strains used in different countries were reported only occasionally in the articles. We included studies for which information about the strain could be obtained from the article itself or from other reliable sources. In all studies in the US, the NYCBH strain was used. In Europe, different strains were used in different countries, regions, and periods. In Germany and Austria in the 1950s and 1960s, the Bern strain was used most often [22]. In the former Soviet Union, the EM-63 strain, which was derived from the NYCBH strain, was used until 1971 [22]. In the UK, the Lister-Elstree strain was developed and used [23]. In the Netherlands, the Copenhagen strain was used until 1962, after which the Lister-Elstree strain was used [24]. In 1968, in an effort to standardize vaccines worldwide and ensure vaccine quality, the WHO recommended that either the NYCBH strain or the Lister strain be used in the worldwide eradication campaign [22]. In both France and Sweden, the Lister strain was used during the times that the data we used here were originally recorded (personal communication with public health officials in the respective national public health institutes). In the former East Germany, the Berlin strain was used, according to information from the Robert Koch Institute (Berlin, Germany). Unknown strains or strains for which too few data were available to make a separate evaluation possible were grouped into the category “other.”

## Methods

Estimating the frequency of rare adverse events from a number of studies presents a methodological problem, because many studies of limited size will report counts of zero. Standard meta-analytic methods, such as those described by Sutton et al. [25], are not well suited to deal with zero counts. We used Bayesian methods to deal with the extremely low but positive probabilities that lead to frequent occurrence of zero counts in the data. Moreover, Bayesian methods provide a natural framework for dealing with a hierarchical model structure and uncertainties. The technical details of our approach can be found in Protocol S1 and Table S1.

**Figure 1:**
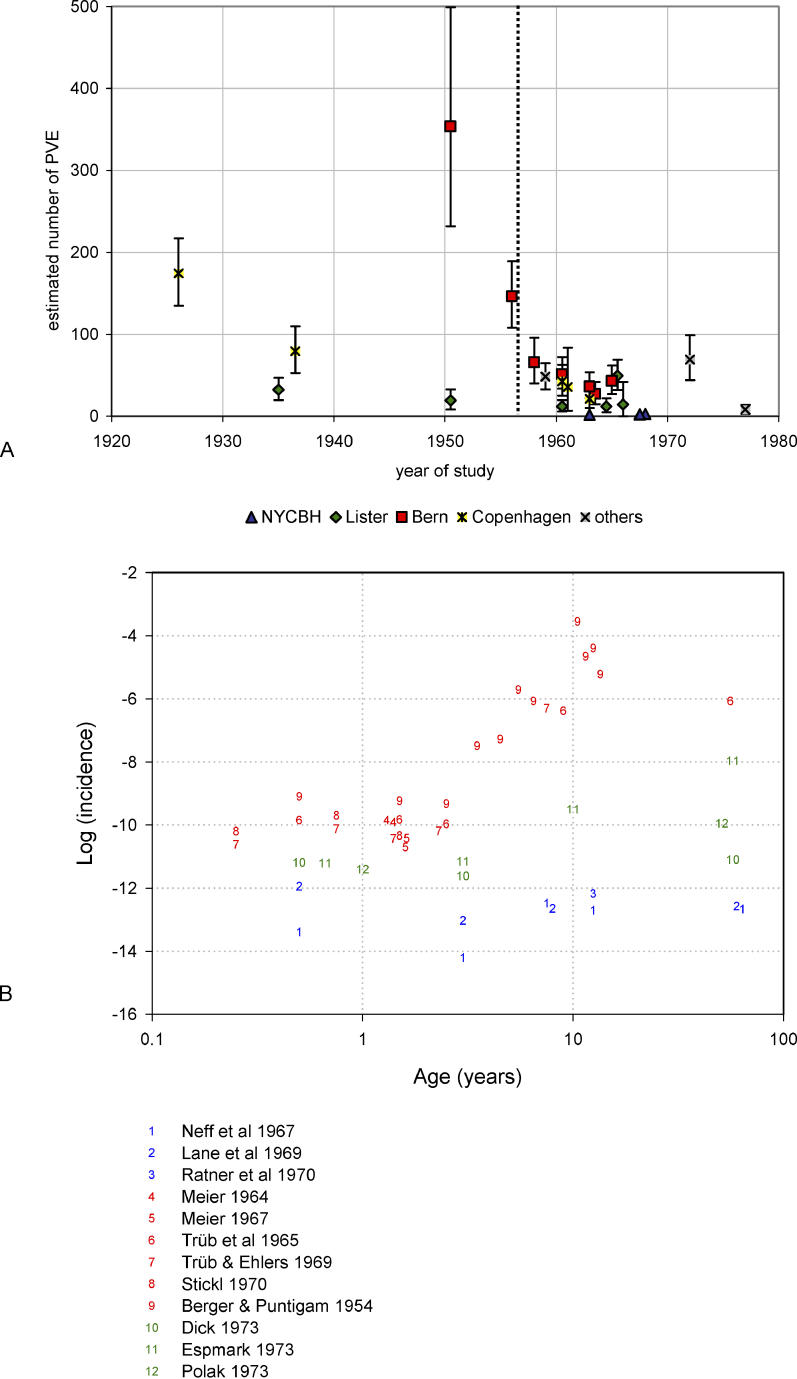
Variability among Studies in the Frequency of PVE (A) The estimated frequency of PVE after primary vaccination in different studies. The symbols mark the midpoint of the interval during which vaccinations reported in the study were performed. The dashed line indicates the separation line between periods that was used for the Bayesian analysis. All studies to the right of the separation line were used for further analysis. The error bars indicate the 95% CIs of the estimates. The data shown in this figure come from the studies marked in the column “time period effect” of Table 1. (B) The logarithm of the incidence of PVE (defined as the number of PVE cases per 1 million vaccinations) by age for different strains, as reported in various studies (blue, NYCBH; red, Bern; green, Lister). The age is plotted on a log_10_ scale for visual clarity.

**Figure 2:**
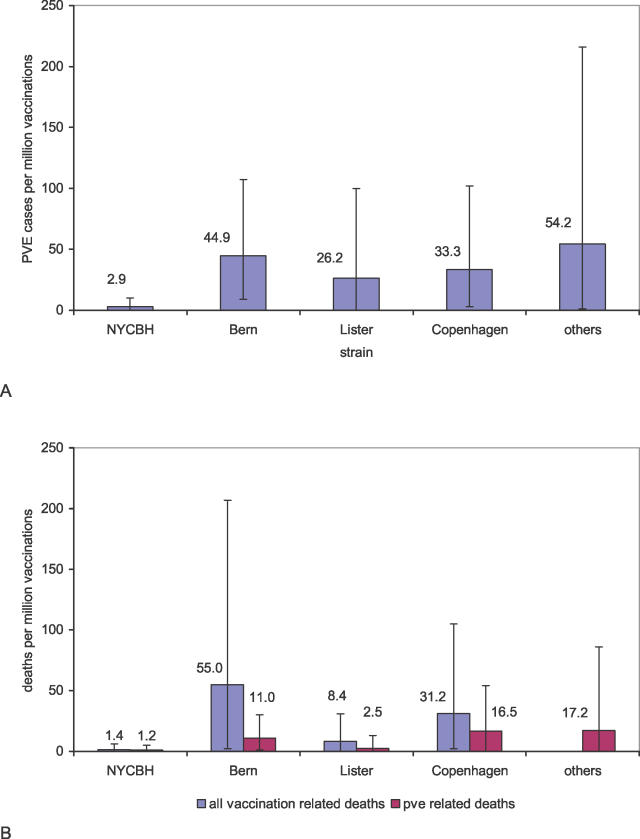
Estimated Frequency of Adverse Events after Vaccination with Different Vaccinia Strains (A) The estimated number of cases of PVE per million primary vaccinations with different vaccinia strains, and (B) the estimated number of deaths (all vaccination-related deaths and PVE-related deaths) per million primary vaccinations with different vaccinia strains. Data in the category “other” are included because they were used in the statistical analysis and therefore, in the Bayesian setting, had an influence on all estimates. The large error bars (95% CIs) indicate the uncertainty of the estimates.

**Figure 3:**
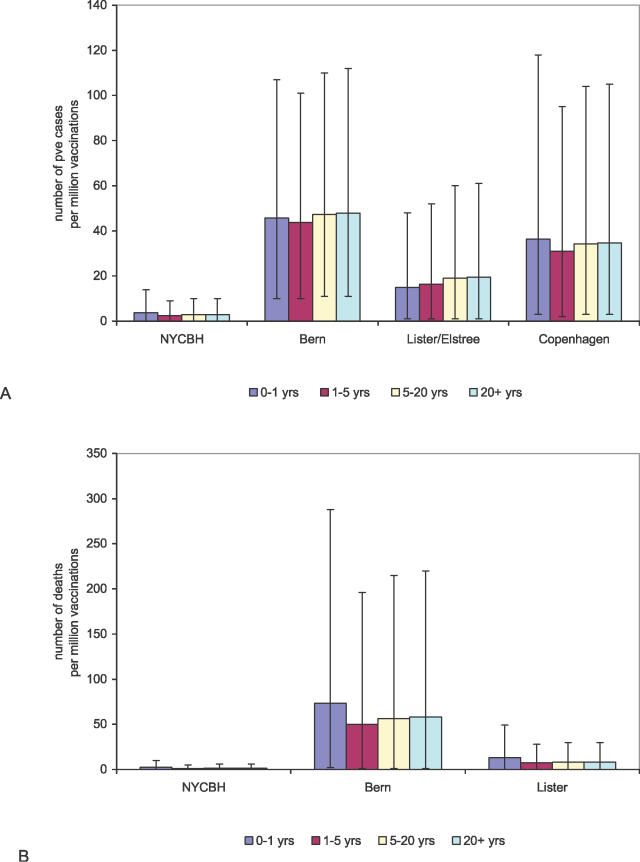
Age-Dependent Estimates for the Frequency of Adverse Events after Vaccination (A) The number of cases of PVE per million vaccinations expected in different age groups for different vaccinia strains, and (B) the number of deaths per million vaccinations expected in different age groups for different vaccinia strains. The large error bars (95% CIs) indicate the uncertainty of the estimates.

**Figure 4:**
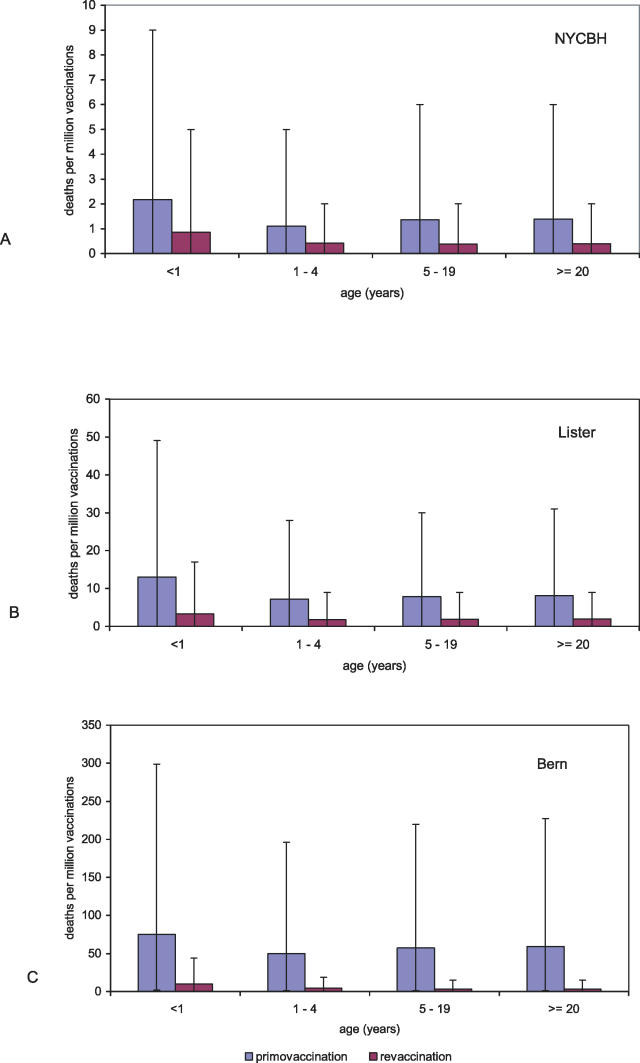
Estimates for the Frequencies of Adverse Events after Primary and Re-Vaccination The expected number of deaths per million primary vaccinations and revaccinations with (A) the NYCBH strain, (B) the Lister strain, and (C) the Bern strain for different age groups. The large error bars (95% CIs) indicate the uncertainty of the estimates.

The analysis was conducted in several steps. First, the effects of the study methodology and the times of data collection on the incidence of PVE were analyzed (Figure 1). In this article, we use the term “incidence” to express number of adverse events per number of vaccinations, without reference to a time unit. We used the midpoint of the reported time of vaccination as a point estimate for the time corresponding to the study data. We estimated the incidences for the different studies with the age-independent hierarchical model (i.e., the model with *ϕ*_*s*_(*a*) ≡ 1; compare Protocol S1). This analysis revealed that earlier studies reported a much higher incidence of adverse events than studies conducted in the 1960s and later (Figure 1A). Therefore, to exclude time trends we used only the more recent studies for the further analysis and estimation. Using data from 1958 onwards, we conducted an analysis with the age-independent model to investigate the effects of different vaccinia strains on the frequency of PVE and death (Figure 2). Using the model with age dependence, we then extended the analysis to study the effects of age and vaccinia strain on the frequency of PVE and mortality (Figure 3). Finally, for the NYCBH, Lister, and Bern strains, age-dependent data about the occurrence of PVE and death after revaccination were analyzed (again, only data from 1958 onwards were used) (Figure 4). Combining primary vaccination and revaccination results with demographic information and information about previous vaccinations in the present population, one can estimate the number of expected cases of PVE and death after mass vaccination with the above vaccinia strains. We provide such estimates for populations in the Netherlands and Germany.

The estimates we report are means of the posterior distribution. Variability of the estimates is described in terms of 95% CIs, which contain 95% of the mass of the density of the posterior distribution. Data in the category “other” were included in the statistical analysis for the sake of completeness, but we do not discuss the findings for these data further because they cannot be interpreted in terms of specific vaccinia strains.

## Results

### Relationship between Study Period and the Reported Incidence of PVE after Primary Vaccination

The estimated incidences of PVE after primary vaccination in studies conducted in different periods showed a clear trend toward lower incidences in more-recent periods (Figure 1A). This was consistent for all strains for which information is available from several decades of reporting. The trend seemed to stabilize in approximately 1960, which led us to take the year 1957 as a cutoff point for distinguishing between earlier and later periods of study. This trend toward lower incidences can be explained by improved vaccine production methods, quality control (as advocated by the WHO), and health-care and vaccination procedures [22]. It could also be a consequence of better case ascertainment that led to a reduced number of adverse events that were misdiagnosed as PVE. It is to be expected that effects associated with present use of a smallpox vaccine will resemble the effects of a similar vaccine administered after 1957. Therefore, in the further analysis, we concentrated on analyzing those studies whose midpoint of observation was no earlier than 1958, the year that marks the introduction of the first WHO standards for smallpox vaccine [22], which followed a study by Cockburn et al. [26].

### Frequency of PVE after Primary Vaccination

For primary vaccinations, we found large differences in the frequency of PVE between vaccine strains (Figure 2A and Table S2). We found that the Bern strain caused by far the highest rate of PVE, with 44.9 expected cases of PVE per million vaccinations, followed by the Copenhagen strain, with 33.3 expected cases per million vaccinations. The Lister strain caused an intermediate rate of PVE, with an expected number of 26.2 cases per million vaccinations, whereas the NYCBH strain was the most benign, with only 2.9 cases per million vaccinations.

With regard to age-related effects, we found different age-dependent patterns of PVE for the various strains (Figure 1B). For the NYCBH strain, the frequency of PVE was highest among persons ≤1 y of age, it decreased to a minimum for persons aged >1–3 y, and it increased again for persons aged >3 y. For the Bern, Lister, and Copenhagen strains, we saw a monotonous increase in the frequency of PVE with increasing age. For vaccination with the NYCBH strain, we determined that there would be approximately four cases per million vaccinations in children younger than 1 y (95% CI, 0–14); for the Lister strain, we expect 15 cases per million vaccinations (95% CI, 1–48); for the Bern strain, we expect 46 cases per million vaccinations (95% CI, 10–107); and for the Copenhagen strain, we expect 36 cases per million vaccinations (95% CI, 3–118) (Figure 3A).

### Vaccination-Related Mortality after Primary Vaccination

A similar pattern emerged for vaccination-related mortality. We analyzed the number of all deaths attributed to vaccination and the number of deaths attributed specifically to PVE. With respect to overall mortality, the Bern strain was again the strain with the highest vaccination-related mortality, with 55.0 deaths per million primary vaccinations (95% CI, 2–207) expected. For the Copenhagen strain, we expect 31.2 deaths per million vaccinations (95% CI, 2–105); for the Lister strain, we expect 8.4 deaths per million vaccinations (95% CI, 0–31); and for the NYCBH strain, we expect only 1.4 deaths per million vaccinations (95% CI, 0–6). Analysis of the number of deaths that are related to PVE only revealed that, for the Bern strain, there would be 11.0 deaths per million vaccinations (95% CI, 1–30); for the Copenhagen strain, there would be 16.5 deaths per million vaccinations (95% CI, 0–54); for the Lister strain, there would be 2.5 deaths per million vaccinations (95% CI, 0–13); and for the NYCBH strain, there would be 1.2 deaths per million vaccinations (95% CI, 0–5) (Figure 2B and Table S2). Combining these estimates with the number of expected cases of PVE leads to estimates of case-fatality rates of 10%–50%. This range indicates the order of magnitude of the case-fatality rate but should not be interpreted as comprising strain-specific estimates in view of the uncertainties involved, such as differences in case ascertainment and case definitions among the studies.

Examination of the age dependence of all vaccination-related mortality reveals a clear pattern for the Bern, Lister, and NYCBH strains. For all three strains, mortality is expected to be highest in children younger than 1 y old and lowest in children approximately 2 y of age. Older children and adults are expected to have a slightly increased mortality rate (Figure 3B). For the other strains, there were not sufficient age-dependent data available to conduct the analysis. After vaccinating children before the age of 1 y, one expects 2.2 deaths per million vaccinations (95% CI, 0–10) when vaccinating with the NYCBH strain, 13.0 deaths per million vaccinations (95% CI, 0–49) when vaccinating with the Lister strain, and 73.3 deaths per million vaccinations (95% CI, 2–288) when vaccinating with the Bern strain. For children aged 1–5 y, one expects 1.1 deaths per million vaccinations (95% CI, 0–5) when vaccinating with the NYCBH strain, 7.3 deaths per million vaccinations (95% CI, 0–28) when vaccinating with the Lister strain, and 50.1 deaths per million vaccinations (95% CI, 1–196) when vaccinating with the Bern strain. The age dependence of mortality was reflected in vaccination schemes for children in Germany and Austria in the 1960s and 1970s, when children were not allowed to receive a primary vaccination after the age of 3 y. The higher levels of mortality in children younger than 1 y of age are taken into account in the present pre-event vaccination guidelines in the US, where age of <1 y is a contraindication for vaccination.

### Mortality after Primary Vaccination and Revaccination

For the NYCBH, Lister, and Bern strains, there are also data about the frequency of death after revaccination. In Figure 4, the expected number of deaths per million vaccinations is shown for different age groups for primary vaccination and revaccination (see also Table S3). In general, one can say that mortality after revaccination is much lower than that after primary vaccination. The age dependence is roughly the same, although for revaccination mortality seems to increase only slightly with increasing age. The differences between strains after revaccination are similar to those after primary vaccination: the NYCBH strain caused the lowest rate of adverse events, the Lister strain caused an intermediate rate, and the Bern strain clearly caused the highest rate.

### Expected Death Toll during a Present-Day Mass Vaccination Campaign

These results can be used to estimate the number of deaths during a mass vaccination campaign for a population with a given population size, age distribution, and vaccination history. We present results for the Netherlands (population, 16 million) and Germany (population, 82 million). Both countries have stockpiled smallpox vaccines based on the Lister strain. We used population age distributions based on recent national demographic data for both countries. We assumed that everybody older than 30 y had been vaccinated before, and that 20% of the population had a contraindication for vaccination. These assumptions are rough approximations because vaccination programs were reduced gradually until they were stopped in the mid-1970s. Under these assumptions, in the Netherlands, mass vaccination with the NYCBH strain would lead to 9.8 deaths per million vaccinations (95% CI, 0–30), mass vaccination with the Lister strain would lead to 55.1 deaths per million vaccinations (95% CI, 7–182), and mass vaccination with the Bern strain would lead to 303.5 deaths per million vaccinations (95% CI, 19–1,093). In Germany, mass vaccination with the NYCBH strain would lead to 46.2 deaths per million vaccinations (95% CI, 6–142), mass vaccination with the Lister strain would lead to 268.5 deaths per million vaccinations (95% CI, 39–875), and mass vaccination with the Bern strain would lead to 1,381 deaths per million vaccinations (95% CI, 94–4,909). These estimates could be refined if precise knowledge of the frequency and distribution of contraindications were available and if more were known about the duration of immunity after vaccination.

## Discussion

Our analysis provides quantitative evidence for the strain-based differences in the occurrence of adverse events described by Fenner et al. [22]. Vaccination with the NYCBH strain is expected to cause a low rate of adverse events, whereas vaccination with the Lister strain is expected to cause an intermediate rate of adverse events, and vaccination with the Bern strain is expected to cause a high rate of adverse events. The frequency of occurrence of adverse events is shown to be age dependent. Combined with demographic data and information about past vaccination schemes, our estimates can be used to provide information about the number of deaths and cases of PVE that are to be expected during a smallpox vaccination campaign. This could be a mass vaccination campaign, or it could be a campaign targeting specific age groups in the population (e.g., schoolchildren and military recruits). Underlying reasons for the observed age dependence might be the maturation of the immune system during childhood and the age-dependent prevalence of certain diseases that impair the immune system and were not recognized in vaccinees at the time of vaccination. A similar age dependence was shown to exist for mortality after smallpox infection [27]. However, the analysis also shows that the support for the age dependency of the data is not strong, and the uncertainties of the estimates, as given in the 95% CIs, are large.

At present, there are efforts to develop second-generation vaccines produced with cell cultures under modern laboratory standards, but these vaccines are not yet licensed [28,29]. As long as they have not been used in large-scale vaccination programs, we cannot know whether they cause fewer adverse events than first-generation vaccines, because the frequency of adverse events is too low to be reliably estimated in Phase 1, Phase 2, and Phase 3 vaccine trials. Therefore, our present knowledge of adverse events after smallpox vaccination is primarily based on what we know from pre-eradication smallpox vaccination programs. Of course, there are many caveats in applying estimates derived from historical data to modern society, many of which have been discussed in the literature [18]. The techniques of vaccine production and quality control have improved [28], and education, training, and screening protocols are implemented with great care [7], such that fewer adverse events might occur. On the other hand, diagnostic techniques and validity have improved, such that it can be decided with improved accuracy whether an observed condition is attributable to smallpox vaccination.

In present vaccination programs, more-rigid screening might be implemented before vaccination to select against persons with a contraindication. On the other hand, the fraction of the population with immune deficiencies, allergies, or other contraindications is thought to be much larger than it was some decades ago, so that even with improved screening the frequency of adverse events might increase. Also, improved diagnostic methods and better surveillance might lead to more-complete reporting and, therefore, an increase in the reported frequency of adverse events. In the recent US vaccination campaign involving military personnel, the fraction of medical exemptions from vaccination was less than 20%, which contrasts with our assumption above. At the same time, the frequency of adverse events was less than expected on the basis of historical data [5]. However, the target population of the program was a very specific population group with a better health status, on average, than the general population.

As the recent vaccination campaign in the US has shown, adverse events may occur that were observed only incidentally in pre-eradication times. During the vaccination campaign conducted in 2003–2004 among civilian volunteers and military personnel, an unexpectedly high frequency of myopericarditis occurred [30,31]. Although the rate of cardiac ischemic events was not higher than expected in the vaccinated population, and the frequency of anticipated adverse events was even lower than expected on the basis of historical records, the unanticipated high frequency of myopericarditis led to negative publicity for the vaccination program and to a plateau in the numbers of vaccinations. It is difficult to assess retrospectively whether some fraction of cardiac deaths in the pre-eradication era could have been attributed to smallpox vaccination but were not recorded as such. In an analysis of death certificates from the period after the mass vaccination campaign in New York City in 1947, Thorpe et al. [32] concluded that an increase in the number of cardiac deaths after vaccination could not be shown. However, their statistical analysis was not undisputed, and the results depended on the underlying assumptions [33]. The recent US vaccination experience emphasizes the importance of accurate surveillance of adverse events after vaccination, combined with accurate statistical analysis of those data. Statistical analysis can provide a basis for defining threshold values and, therefore, tools for deciding about continuation or changes in vaccination programs. In particular, it might help in weighing the advantages and disadvantages of ring vaccination against those of mass vaccination. Such a decision, however, will include more than numerical estimates; that is, it will also have to take into account what is politically and socially acceptable at the moment of implementing the vaccination program. More-detailed information about the proportion of persons vaccinated in every age group, the duration of immunity after vaccination, and the proportion of persons in each age group with a contraindication is necessary for a more precise calculation of the frequencies of adverse events expected in specific populations.

A limitation of our analysis lies in the variable quality of the historical data, which cannot be checked for reliability retrospectively. The data reported in different studies could be based on different methods of case ascertainment and different case definitions. The implementation of vaccination programs, pre-vaccination screening, and the vaccination methods might have been different in different countries. Also, there are large differences in the surveillance and reporting of adverse events. We found a clear time trend in the occurrence of adverse events that reflected improvements in quality control and standardization of vaccine production as advocated by the WHO [22,26]. But intensified screening for contraindication toward the end of the eradication program might also have contributed to decreased incidences. The Bayesian methods used here offer an opportunity to take some of the heterogeneities into account by including the effect of study methodology in the statistical analysis. Therefore, Bayesian methods allow a modern statistical analysis of these extensive historical datasets.

Our analysis shows that the frequency of serious adverse effects of smallpox vaccination is considerably higher for the Lister strain than for the NYCBH strain. Previous analyses discussing vaccination as a response to a bioterrorist attack with smallpox [34,35] have been based almost exclusively on estimates for the NYCBH strain obtained from the national surveillance study of Lane et al. [14]. Furthermore, in modeling studies, such as those published by Kaplan et al. [10], Halloran et al. [9], and Porco et al. [13], the number of deaths after vaccination was estimated as one per million vaccinations and was independent of the ages of the individuals vaccinated. Our analysis places these estimates on the lower end of a range of estimates for the NYCBH strain and shows that estimates for the Lister strain are even considerably higher. This implies that estimates based on the NYCBH strain cannot easily be used to estimate the effects of vaccination in European countries, where most of the existing stockpiles of smallpox vaccine are based on the Lister strain. On the basis of our age-specific estimates, country-specific estimates can be derived for the expected number of deaths after vaccination for each vaccinia strain, the age distribution of the target population, and the population's prevaccination history. Policy makers planning for an emergency response in case of a smallpox outbreak in Europe should be aware of the increased risk of adverse events associated with vaccination with the Lister strain, compared with the NYCBH strain.

## Supporting Information

Protocol S1 Statistical Methods (31 KB DOC)

Table S1 Estimates for the Parameters Determining the Age-Dependence of Adverse Effects The mean estimates are given for the parameters *βs*, *σs*, and *ρs* for the parameters of *φs(a)* describing the age-dependence of the occurrence of PVE and mortality.
(34 KB DOC)

Table S2 Expected Cases of PVE, Deaths Related to PVE, and All Deaths per 1 Million Primary Vaccinations for Different Vaccinia Strains 95% CIs are given in brackets (shown in Figure 2).
(32 KB DOC)

Table S3 Expected Number of Deaths after Primary Vaccination and Revaccination with NYCBH, Lister, and Bern Vaccinia Strains for Different Age Groups 95% CIs are given in brackets (shown in Figure 4).
(35 KB DOC)

## Abbreviations

CI: credible interval
NYCBH: New York City Board of Health
PVE: postvaccinal encephalitis
WHO: World Health Organization
