# Correction: Frequency of Adverse Events after Vaccination with Different Vaccinia Strains

**DOI:** 10.1371/journal.pmed.0030429

**Published:** 2006-10-31

**Authors:** Mirjam Kretzschmar, Jacco Wallinga, Peter Teunis, Shuqin Xing, Rafael Mikolajczyk

In *PLoS Medicine*, volume 3, issue 8: DOI: 10.1371/journal.pmed.0030272

Under the section heading “Expected Death Toll during a Present-Day Mass Vaccination Campaign,” the numbers of expected deaths that were computed for a mass vaccination campaign were displayed as per million; they should be absolute numbers.

The corrected text should be: “Under those assumptions, in the Netherlands mass vaccination with NYCBH would lead to 9.8 deaths (95% CI [0, 30]), mass vaccination with Lister to 55.1 deaths (95% CI [7, 182]), and mass vaccination with Bern would lead to 303.5 deaths (95% CI [19, 1093]). In Germany mass vaccination with NYCBH would lead to 46.2 deaths (95% CI [6, 142]), mass vaccination with Lister would lead to 268.5 deaths (95% CI [39, 875]), and mass vaccination with Bern would lead to 1,381 deaths (95% CI [94, 4909]).”

