# Supplementary material for: Frequency of Adverse Events after Vaccination with Different Vaccinia Strains
Source: PLoS Med. 2006 Aug 22;3(8):e272. doi: 10.1371/journal.pmed.0030272 (PMC1551910; doi:10.1371/journal.pmed.0030272)
Supplement: Supplementary file 1 [file pmed.0030272.sd001.doc]

### Supporting information

### Statistical methods

We denote by *y* the number adverse events given *N* vaccinations with strain *s* in the age interval *[l, u].* We have *n* observations *Y_i_ = (y_i_, N_i_, s_i_, l_i_, u_i_), i=1,...n*, and want to estimate parameters *Θ = (θ_1_,..., θ_n_)* that describe the probability of occurrence of an adverse event given vaccination at a certain age with a certain strain. The observed number of adverse events is a binomial sample from a population *N_i_* with proportion *θ_i_*. Since the probability of adverse events is very small we approximate the binomial by a Poisson distribution for all *i*. The likelihood function for *Y* is then given by

$L(Y|\Theta)=\prod_{i=1}^{n} f(y_{i}|\theta_{i}N_{i})$ (1)

where *f* is a Poisson distribution with mean *θ_i_ N_i_*. We assume that the probability *θ* of an adverse event is the product of a vaccinia strain dependent factor *ζ_s_* and an strain- and age-dependent factor *φ_s_(a)*, i.e. $\theta=\zeta_{s}\phi_{s}(a)$, subject to the constraint that this product is restricted to the interval [0, 1]. If $\phi_{s}(a)\equiv1$, the model is independent of age. As a prior for *ζ_s_* we take a gamma distribution with strain dependent parameters *γ* and *δ* for scale and shape:$\zeta_{s}\sim Gamma(\gamma(s),\delta(s))$. In addition, to take the variability between studies into account, the parameters *γ* and *δ* are dependent on the study (for reasons of simplicity this is not included as an extra index in the notation). The priors for the parameters *γ* and *δ* are chosen such that *log γ* and the log mean of the gamma distribution (log (γδ)) are normally distributed.

To describe the age effect we define the function *φ_s_* as

$\phi_{s}(a)=\exp(-\beta_{s}a)+\frac{\sigma_{s}a}{a+\rho_{s}}$ (2)

with strain dependent parameters *β_s_*, *σ_s_*, and *ρ_s_* that have gamma distributed priors to ensure that their values are positive. With the function *φ* the effect of age is normalized to 1 at age zero. Furthermore the function is the sum of a decreasing exponential function and an increasing logistic function with an asymptote at *σ_s_*. Depending on the quantitative relationship of the three parameters, *φ* might decrease or increase for younger ages. For older ages, it converges to a constant value. In the estimation procedure, the cumulative number of cases of an adverse effect is evaluated at the upper age bound, i.e. we consider the number of cases that occur when vaccination takes place at an age younger than the upper age bound. We analyzed the model using the WinBUGS software developed by the MRC and Imperial College, UK (<http://www.mrc-bsu.cam.ac.uk/bugs>).
