# Supplementary material for: Frequency of Adverse Events after Vaccination with Different Vaccinia Strains
Source: PLoS Med. 2006 Aug 22;3(8):e272. doi: 10.1371/journal.pmed.0030272 (PMC1551910; doi:10.1371/journal.pmed.0030272)
Supplement: Supplementary file 2 [file pmed.0030272.st001.doc]

Table S1: The mean estimates are given for the parameters *β_s_*, *σ_s_*, and *ρ_s_* for the parameters of *φ_s_(a)* describing the age-dependence of the occurrence of PVE and mortality.

|  | | *NYCBH* | *Bern* | *Lister* | *Copenhagen* | *others* |
| --- | --- | --- | --- | --- | --- | --- |
| *PVE* | *β_s_* | 1.087 | 1.03 | 0.8649 | 1.171 | 2.18 |
|  | *σ_s_* | 1.003 | 1.618 | 1.694 | 1.451 | 1.926 |
|  | *ρ_s_* | 0.8328 | 0.7162 | 1.551 | 0.844 | 1.218 |
| *Mortality* | *β_s_* | 1.084 | 0.9865 | 0.7016 | 1.046 | 0.9394 |
|  | *σ_s_* | 0.8085 | 0.9133 | 0.7001 | 1.337 | 1.097 |
|  | *ρ_s_* | 0.9666 | 0.9887 | 0.4715 | 0.8088 | 0.9699 |
