# Supplementary material for: Frequency of Adverse Events after Vaccination with Different Vaccinia Strains
Source: PLoS Med. 2006 Aug 22;3(8):e272. doi: 10.1371/journal.pmed.0030272 (PMC1551910; doi:10.1371/journal.pmed.0030272)
Supplement: Supplementary file 3 [file pmed.0030272.st002.doc]

Table S2: Expected cases of PVE, deaths related to PVE, and all deaths per one million primary vaccinations for different vaccinia strains. In bracket the 95% credible intervals are given (shown in Figure 2).

|  | NYCBH | Lister | Bern | Copenhagen | others |
| --- | --- | --- | --- | --- | --- |
| PVE cases | 2.9  [0, 10] | 26.2  [0, 100] | 44.9  [9, 107] | 33.3  [3, 102] | 54.2  [1, 216] |
| PVE deaths | 1.2  [0, 5] | 2.5  [0, 13] | 11.0  [1, 30] | 16.5  [0, 54] | 17.2  [0, 86] |
| all deaths | 1.4  [0, 6] | 8.4  [0, 31] | 55.0  [2, 207] | 31.2  [2, 105] | -^[1]^ |

1. No data available
