# Supplementary material for: Frequency of Adverse Events after Vaccination with Different Vaccinia Strains
Source: PLoS Med. 2006 Aug 22;3(8):e272. doi: 10.1371/journal.pmed.0030272 (PMC1551910; doi:10.1371/journal.pmed.0030272)
Supplement: Supplementary file 4 [file pmed.0030272.st003.doc]

Table S3: Expected number of deaths after primary and revaccination with NYCBH, Lister, and Bern for different age groups. In brackets the 95% credible intervals are given (shown in Figure 4).

|  | NYCBH | | Lister | | Bern | |
| --- | --- | --- | --- | --- | --- | --- |
| age (yrs) | primovacc | revacc | primovacc | revacc | primovacc | revacc |
| 0-1 | 2.2  [0, 10] | 0.9  [0, 5] | 13.0  [0, 49] | 3.3  [0, 17] | 73.3  [2, 288] | 9.7  [0, 44] |
| 1-5 | 1.1  [0, 5] | 0.4  [0, 2] | 7.3  [0, 28] | 1.8  [0, 9] | 50.2  [1, 196] | 4.5  [0, 19] |
| 5-20 | 1.4  [0, 6] | 0.4  [0, 2] | 7.8  [0, 30] | 1.9  [0, 9] | 56.5  [1, 215] | 3.5  [0, 15] |
| 20+ | 1.4  [0, 6] | 0.4  [0, 2] | 8.1  [0, 30] | 2.0  [0, 9] | 58.0  [1, 220] | 3.5  [0, 15] |
